# Biphasic *Slc2a4* Gene Expression in 3T3-L1 Adipocytes in Response to Treatment with Low and High Concentrations of Daidzein and Genistein

**DOI:** 10.3390/cimb47100857

**Published:** 2025-10-17

**Authors:** Karen Cristina Rego Gregorio, Caroline Pancera Laurindo, Helayne Soares Freitas, Maristela Mitiko Okamoto, Patricia Monteiro Seraphim, Ubiratan Fabres Machado

**Affiliations:** 1 Department of Physiology and Biophysics, Institute of Biomedical Sciences, University of São Paulo, São Paulo 05508-000, SP, Brazil; karengregoriorego@gmail.com (K.C.R.G.); panceracaroline@gmail.com (C.P.L.); hfreitas2@gmail.com (H.S.F.); maristelamitiko@gmail.com (M.M.O.); 2 Graduate Program in Movement Sciences, Department of Physiotherapy, Faculty of Sciences and Technology, Sao Paulo State University (UNESP), Campus (Presidente Prudente), Presidente Prudente 19060-900, SP, Brazil; pm.seraphim@unesp.br

**Keywords:** diabetes mellitus, obesity, GLUT4, isoflavones, phytoestrogens, estrogen, 17β-estradiol, estrogen receptor, ESR1, ESR2

## Abstract

Daidzein and genistein are abundant in soy-rich foods, whose supplementation has been proposed to have beneficial effects on several diseases, including diabetes mellitus and obesity. 17β-estradiol (E2) enhances the expression of the *Slc2a4* gene and GLUT4 protein in adipose tissue, increasing glucose consumption and contributing to glycemic control. We investigated, in 3T3-L1 adipocytes, the effect of low and high doses of daidzein and genistein on *Slc2a4*/GLUT4 expression and the participation of estrogen receptors 1/2 (ESR1/ESR2) in the regulations observed. Differentiated adipocytes were cultivated, for 24 h, in the presence of 17β-estradiol (E2, 10 nM), daidzein (10 nM–150 μM) and genistein (10 nM–50 μM), with or without ESR1 or ESR2 antagonists. Daidzein/genistein at a low dose (10 nM) increased *Slc2a4*/GLUT4 expression (50%, *p* < 0.05), an effect abrogated by an ESR1 antagonist, mimicking the effect of E2. However, maximal doses of daidzein and genistein reduced, in a ESR1-mediated mechanism, the expression of mRNA (by 47% and 60%, *p* < 0.001) and the protein (by 29% and 36%, *p* < 0.01), respectively, for daidzein and genistein, as compared to E2. In conclusion, in adipocytes, daidzein and genistein have a biphasic ESR1-mediated effect: while low concentrations increase the expression of *Slc2a4*/GLUT4, high concentrations decrease it, the former predisposing to an adipogenic effect, the latter to a diabetogenic condition.

## 1. Introduction

Improvement of glycemic control by estrogenic substances in women with diabetes mellitus (DM) dates to a century ago [1]. Since then, impaired glycemic control, related to insulin resistance, has been described in women under hypoestrogenic conditions, worsening DM states [2,3]; however, insulin resistance and increased incidence of DM have also been related to hyperestrogenism [4]. Similarly, in men, in whom estrogens also regulate reproductive and nonreproductive biological systems [5], both decreased and increased estrogenic activities have also been related to insulin resistance [6,7,8]. Thus, the role of estrogen hormones in glycemic homeostasis seems to be complex, suggesting the participation of still unknown mechanisms, which might explain the apparent controversial modulations.

Estrogen actions are mainly mediated by nuclear estrogen receptors 1 and 2 (ESR1 and ESR2, former ERα and ERβ, respectively), which bind to an estrogen receptor element (ERE) in target genes, thus modulating a transcriptional activity that can either activate or repress gene expression (for a review, see [9,10,11]). Concerning the mechanisms of estrogen action in glycemic homeostasis regulation, remarkable studies have demonstrated that ESR1 activity increases the expression of the *Slc2a4* gene (solute carrier family 2 member 4), which codifies the GLUT4 protein (solute carrier family 2, facilitated glucose transporter member 4), whereas ESR2 activity decreases it [12,13,14]. Regarding this, it is important to point out that GLUT4 is a specific glucose transporter mainly expressed in tissues that take up glucose in response to insulin (adipose and skeletal muscle tissues), playing a fundamental role in the postprandial glycemic homeostasis [15]. In fact, the 24 h treatment of 3T3L1 adipocytes with physiological doses of 17β-estradiol (E2) can increase the expression of *Slc2a4*/GLUT4, as well as the translocation of GLUT4 to the plasma membrane, in an ESR1-mediated way [12,16]. Notably, these effects lead to an increase in cellular glucose uptake [16], thus revealing a mechanism through which estrogen could improve glycemic homeostasis, although it could also predispose adipogenesis.

Among phytoestrogens, molecules capable of interacting with ESRs, the isoflavones daidzein and genistein have high estrogenic potency and are very abundant in some foods [17]. The actions of isoflavones are rather complex, since they are related to many factors not clearly identified yet. Despite this, isoflavones come into focus due to their increasingly positive effects on human health [18], including glycemic homeostasis modulation [19]. Isoflavones can bind with a variable affinity to both ESR1 and ESR2; however, it has been widely accepted that they more strongly bind to ESR2 [20,21]. After binding to ESRs, isoflavones can induce a variable degree of activation/inhibition of target genes, thus mimicking estrogen effects [21,22]. Nevertheless, they may block the estrogen receptor ligand-binding domain, thus blocking estrogenic activity, even in the presence of the hormone [21,22]. All of this reveals that phytoestrogens may induce estrogenic and anti-estrogenic effects.

The current trend of phytoestrogen intake, particularly isoflavones, is a cause of concern regarding glycemic homeostasis regulation [19]. Considering that ESR2-mediated E2 activity produces a diabetogenic effect [13], and considering that isoflavones more strongly bind to ESR2 [20,21], we might expect that the consumption of isoflavones could have the worrisome potential to deteriorate glycemic homeostasis, an effect which would involve the repression of *Slc2a4*/GLUT4 expression. Thus, the present study was de-signed to investigate the effect of low and high doses of daidzein and genistein on *Slc2a4*/GLUT4 expression by adipocytes in culture, and to analyze the participation of ESR1 and ESR2 in the *Slc2a4* mRNA regulations observed. The results revealed that isoflavones induce, in an ESR1-mediated way, a biphasic effect on *Slc2a4*/GLUT4 expression in adipose cells, with high concentrations inhibiting the expression of the glucose transporter, thus revealing a potential diabetogenic effect.

## 2. Materials and Methods

### 2.1. Cell Culture and Treatments

Mouse 3T3-L1 preadipocytes were obtained from the American Type Culture Collection, Rio de Janeiro Cell Bank, Rio de Janeiro, Brazil (ATCC^®^ Number: CL-173TM), and cultured as previously described [14,16]. Briefly, the cells were induced to differentiate using DMEM (Dulbecco’s modified Eagle medium, Vitrocell Embriolife, Campinas, SP, Brazil) and 10% FBS (fetal bovine serum; Vitrocell Embriolife, Campinas, SP, Brazil) supplemented with 10 μg/mL regular insulin, 1 μM dexamethasone, and 0.5 mM 3-isobutyl-1-methylxanthine (Sigma-Aldrich; St. Louis, MO, USA) for 8 days, followed by 2 days without supplementation. On day 10, cells were transferred to DMEM with 5.5 mM glucose, 10% FBS, 1% antibiotics (penicillin/streptomycin) (Vitrocell Embriolife, Campinas, SP, Brazil), and 1.5 nM insulin ((Sigma-Aldrich; St. Louis, MO, USA); thus, 24 h treatments were performed with 10 nM of water-soluble 17β-estradiol (E2) (E4389, Sigma-Aldrich, St. Louis, MO, USA); 10 nM, 50 μM or 150 μM daidzein (D) (7802, Sigma-Aldrich, St. Louis, MO, USA); 10 nM, 5 μM or 50 μM genistein (G) (G6649, Sigma-Aldrich, St. Louis, MO, USA), added or not with 1 μM of a selective ESR1 antagonist MPP (M) [1,3-bis(4-hydroxyphenyl)-4-methyl-5-(4-(2-piperidinylethoxy)phenol)-1H-pyrazole dihydrochloride; Tocris, Avonmouth, Bristol, UK]; or 1 μM of a selective ESR2 antagonist PHTPP (P) [(2-Phenyl-5,7-bis(trifluoromethyl)pyrazolo[1,5-a]pyrimidin-3-yl)phenol; Tocris, Avonmouth, Bristol, UK]. Genistein and PHTPP were solubilized in dimethylsulfoxide (DMSO, Merck Millipore, Darmstadt, Germany, #102952), which was added at the same final concentration (0.05%) in daidzein- and MPP-treated cells, as well as in the control (C) cells cultivated without any compound. At the end of the treatments, the cells were subjected to total RNA or protein extraction, as described below. To check possible cytotoxic effects of the high doses of D and G, batches of cell culture dishes, after the 24 h treatments, were additionally treated with yellow tetrazolium salt 3-(4,5-dimethylthiazol-2-yl)-2,5-diphenyltetrazolium bromide (MTT), using the Cell Proliferation Kit-MTT (#11465007001, Merck Millipore, Darmstadt, Germany), according to the manufacturer’s instructions.

### 2.2. Experimental Design

The experimental groups include control cells with no treatment (C) and cells treated with 10 nM 17β-estradiol (E2); 10 nM daidzein (D10nM); 10 nM genistein (G10nM); 10 nM E2 + 10 nM D (E2/D10nM); 10 nM E2 + 10 nM G (E2/G10nM); 1 µM ESR1 antagonist MPP (M); 1 µM ESR2 antagonist PHTPP (P); 10 nM E2 + MPP (E2/M); 10 nM E2 + P (E2/P); 10 nM D + M (D10nM/M); 10 nM D + P (D10nM/P); 10 nM G + M (G10nM/M); 10 nM G + P (G10nM/P); 50 µM D (D50µM); 150 µM D (D150µM); 5 µM G (G5µM); 50 µM G (G50µM); 150 µM D + MPP (D150µM/M); 50 µM G + MPP (G50µM/M); 150 µM D + PHTPP (D150µM/P); or 50 µM G + PHTPP (G50µM/P).

To investigate the effects of different concentrations of D and G, experiments were carried out in three stages: (1) C, 10nME, 10nMD and 10nMG, to analyze equimolar low concentrations; (2) C, 10nME, 50µMD and 5µMG, to analyze high concentrations; and (3) C, 10nME, 150µMD and 50µMG, to analyze even higher concentrations of D and G.

To assess cell viability, four different batches of experiments were conducted in different days, each one containing one culture dish for each experimental condition. In other results, data presented in each graph (four groups) were obtained from at least three different batches of cell culture plates treated in different days, each batch containing three culture plates of six wells (four wells per treatment), achieving at least twelve wells per treatment. For preparation of one sample of mRNA or protein, at the end of treatments, cells of at least two wells were sampled and thus processed.

### 2.3. Reverse Transcription and Quantitative Polymerase Chain Reaction (RT-qPCR)

For mRNA analysis, total RNA from adipocytes was extracted using TRIzol^®^ Reagent according to manufacturer recommendations (Invitrogen, Carlsbad, CA, USA); reverse transcriptase reaction was performed using random primers (Invitrogen, Carlsbad, CA, USA) and the ImProm-II^®^ Reverse Transcription System (Promega Corporation, Madison, WI, USA). Quantitative PCR amplification was performed with PowerUp^®^ SYBR^®^ Green Master Mix (Applied Biosystems Inc., Foster City, CA, USA) and a StepOne Plus Instrument (Applied Biosystems Inc., Foster City, CA, USA). Inventoried probes (Applied Biosystems) were used for mouse *Slc2a4* (Mm01245502_m1) and *Gapdh* (Mm99999915_g1) genes. Relative values of *Slc2a4* mRNA expression were calculated from the threshold cycle (Ct) following the 2^−ΔΔCt^ method; *Gapdh* mRNA was chosen as a reference gene after previous RefFinder algorithm analysis testing of *Gapdh*, Atp5b and Actb genes. Results were expressed as arbitrary units (AU) related to the mean of the controls, set to 1.0.

### 2.4. Western Blotting

For the protein expression measurement, adipocytes were processed to obtain the total cellular membrane protein fraction [23]; total protein content in the samples was quantified using the Bradford method according to manufacturer recommendations (Bio-Rad Laboratories; Hercules, CA, USA). Equal amounts (20 µg) of protein were electrophoresed, transferred into the nitrocellulose membrane and stained with Ponceau for normalizing the results. Membranes were immunoblotted with anti-GLUT4 (#07-1404, Milipore, Burlington, MA, USA, #071404, 1:3000), followed by the appropriate secondary conjugated antibody. The enhanced chemiluminescence (ECL) procedure was performed using the SuperSignal^®^ West Pico Plus Chemiluminescent Substrate (Thermo Scientific, Rockford, IL, USA). The images were taken using the Syngene automated system model G:BOX Chemi XRQ (Synoptics, CA, Cambridge, UK) and the optical density of the blots was analyzed using ImageJ software (National Institutes of Health, Bethesda, MD, USA, version 1.51). The optical density of the respective lane of the Ponceau-stained membrane was used for normalizing the GLUT4 values, expressed as arbitrary units (AU) related to the mean of the controls, which was set to 1.0. Images of the original experiments from which the representative images of GLUT4 were selected, showing the entire Ponceau-stained membrane and the immunoblotted membrane, are presented in Supplementary Figures S1 and S2, as informed in the Results.

### 2.5. Statistical Analysis

Data were expressed as mean ± standard deviation (SD) of 5 to 10 samples, from at least three different batches of experiments, as specified in the legends. Comparisons among the groups were performed by one-way analysis of variance (ANOVA), followed by the Tukey test, after testing the normality of the data distribution by the Shapiro–Wilk test. Analyses were performed using GraphPad Prism (version 10.4.2).

## 3. Results

### 3.1. Low to High Concentrations of Phytoestrogens Do Not Alter Adipocyte Viability in Culture

**Figure 1:**
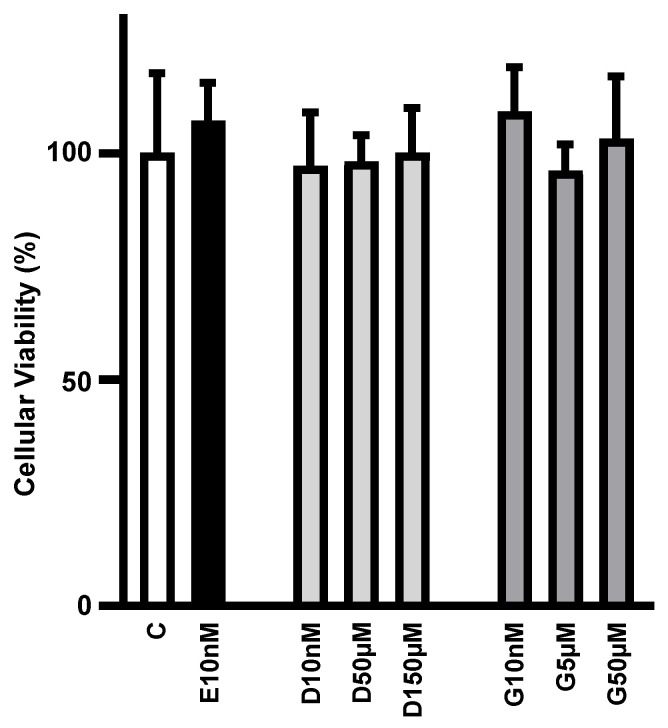
Low to high concentrations of phytoestrogens do not alter adipocyte viability in culture. Cellular viability of differentiated 3T3L1 adipocytes cultivated, for 24 h, with 10 nM of 17β-estradiol (E10nM); 10 nM, D50µM and 150 µM of daidzein (D10nM, D50µM and D150µM); and 10 nM, 5 µM and 50 µM genistein (G10nM, G5µM and G50µM) was analyzed by tetrazolium salt method (MTT) and expressed as % of the mean of the control (C) treatment without hormone. Data are expressed as mean ± SD of four different culture dishes. No significant differences were observed among the groups.

Initially, we investigated whether increasing concentrations of daidzein and genistein could compromise cell viability. Figure 1 shows that cell viability was unaltered in 3T3L1 adipocytes cultivated for 24 h with 17β-estradiol (E2; 10 nM), daidzein (D; 10 nM, 50 µM and 150 µM) and genistein (G; 10 nM, 5 µM and 50 µM), as compared to control cells.

### 3.2. Low Concentration of Daidzein and Genistein Mimics Estrogen’s Enhancing Effect on Slc2a4/GLUT4 Expression in Adipocytes

**Figure 2:**
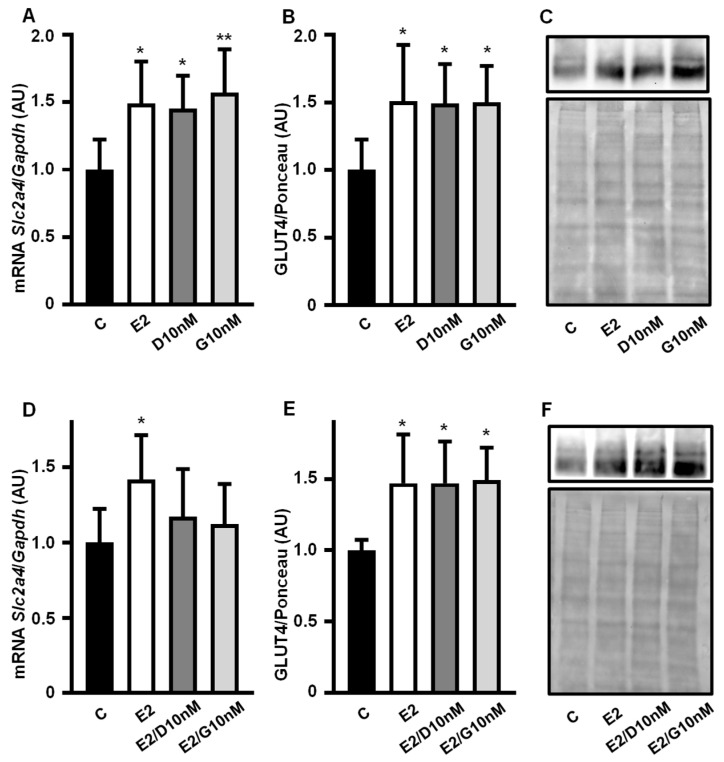
Low concentration of daidzein and genistein mimics estrogen’s enhancing effect on *Slc2a4*/GLUT4 expression in adipocytes. *Slc2a4* mRNA (**A**,**D**) and GLUT4 protein (**B**,**C**,**E**,**F**) expression were analyzed in differentiated 3T3-L1 adipocytes cultivated, for 24 h, with 17β-estradiol, daidzein and genistein, at equimolar concentrations (10 nM), and compared with a control treatment without hormone for 24 h. The *Slc2a4* mRNA was analyzed by RT-qPCR, using the *Gapdh* mRNA as a loading control; the GLUT4 protein was quantified by Western blotting, using the Ponceau-stained membrane as a loading control. Representative blots of GLUT4 and respective Ponceau-stained membrane are shown in (**C**,**F**). Results were normalized considering the mean of C values as 1.0. Data are expressed as mean ± SD of 8 (C groups) and 6 (treated groups) samples, from at least 3 different batches of experiments. The means were compared by one-way ANOVA, followed by Tukey’s multiple comparison test (* *p* < 0.05, ** *p* < 0.01 vs. C). C, control; E2, 17β-estradiol; D, daidzein; G, genistein; AU, arbitrary units; *Slc2a4*, solute carrier family 2 member 4; *Gapdh*, glyceraldehyde-3-phosphate dehydrogenase; GLUT4, solute carrier family 2, facilitated glucose transporter member 4.

Daidzein (10 nM) and genistein (10 nM) increased the *Slc2a4* mRNA (Figure 2A) and GLUT4 protein (Figure 2B,C) expression by ~50% (*p* < 0.05 vs. C), an effect similar to that of equimolar concentrations of E2 (10 nM). Since isoflavones can show different effects in the presence or absence of E2, the addition of E2 to daidzein and genistein was also evaluated. The increase in the *Slc2a4* mRNA expression observed with E2, D and G alone was not observed in response to E2/D and E2/G (Figure 2D); however, the GLUT4 expression (Figure 2E,F) in response to E2/D and E2/G remained the same as that when they were alone.

Images of the original experiments from which the representative images of GLUT4 were selected (Figure 2C,F), showing the entire Ponceau-stained membrane and the immunoblotted membrane, are presented as Supplementary Figure S1.

### 3.3. The Enhancing Effect of Low Concentration of Daidzein and Genistein on Slc2a4 Gene Expression in Adipocytes Involves the Activation of ESR1

**Figure 3:**
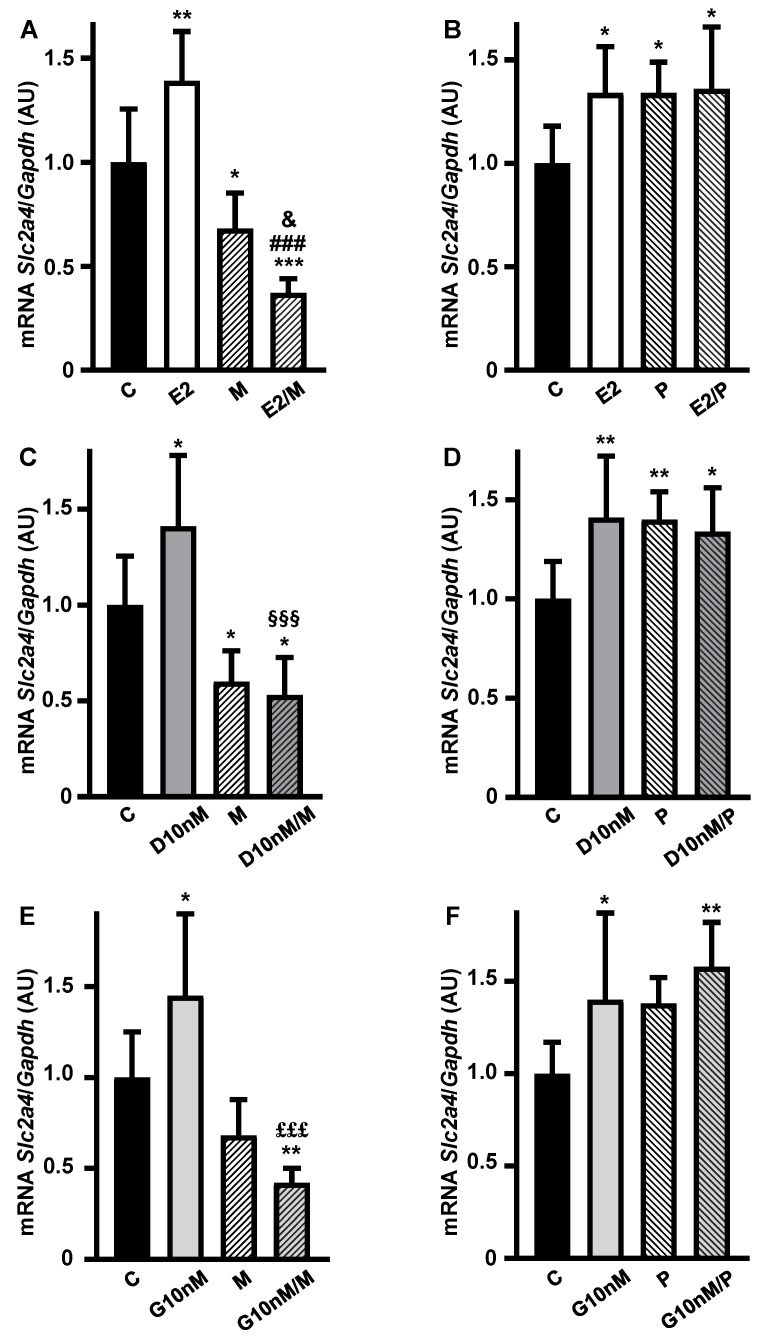
The enhancer effect of low concentrations of daidzein and genistein on the *Slc2a4* gene expression in adipocytes involves the activation of estrogen receptor 1 (ESR1). *Slc2a4* mRNA expression was analyzed in differentiated 3T3-L1 adipocytes cultivated, for 24 h, with 17β-estradiol (**A**,**B**), daidzein (**C**,**D**) and genistein (**E**,**F**), at equimolar concentrations (10 nM), combined or not with 1 µM ESR1 antagonist MPP (1,3-Bis(4-hydroxyphenyl)-4-methyl-5-[4-(piperidinylethoxy)phenol]-1H-pyrazol dihydrochloride) (**A**,**C**,**E**) or 1 µM ESR2 antagonist PHTPP (4-[2-Phenyl-5,7-bis(trifluoromethyl)pyrazolo[1,5-a]pyrimidin-3-yl]phenol) (**B**,**D**,**F**), and compared with a control treatment without hormone. *Slc2a4* mRNA was analyzed by RT-qPCR, using the *Gapdh* mRNA as a loading control. Data are expressed as mean ± SD of 10 (E2P group), 8 (C groups), 6 or 7 (the other groups) samples, from at least 3 different batches of experiments. The means were compared by one-way ANOVA, followed by Tukey’s multiple comparison test (* *p* < 0.05 vs. C, ** *p <* 0.01, *** *p* < 0.001 vs. C; ### *p* < 0.001 vs. E2; & *p* < 0.05 vs. M; §§§ *p* < 0.001 vs. D; £££ *p* < 0.001 vs. G). C, control; E2, 17β-estradiol; D, daidzein; G, genistein; M, ESR1 antagonist MPP; P, ESR2 antagonist PHTPP; AU, arbitrary units; *Slc2a4*, solute carrier family 2 member 4; *Gapdh*, glyceraldehyde-3-phosphate dehydrogenase.

Aiming to investigate the participation of ESRs in the transcriptional enhancer role of E2, D and G on *Slc2a4* expression, the effects of the ESR1- and ESR2-selective antagonists MPP (M) and PHTPP (P), respectively, were analyzed. Firstly, the treatment with M alone showed a reduction (by ~32%, *p* < 0.01 vs. C) in *Slc2a4* mRNA (Figure 3A,C,E), evincing the constitutive enhancer effect of ESR1 on this gene. As expected, the E2 increase in *Slc2a4* mRNA was completely abolished by combining E2/M and the reduction in *Slc2a4* mRNA was greater than that observed with M alone (Figure 3A). Finally, the combination E2/P had no effect on the E2 enhancer effect (Figure 3B), indicating that ESR2 does not participate in the E2-induced stimulation of *Slc2a4* gene expression.

Similar results were observed in response to ESR1 and ESR2 antagonism during the treatments with daidzein (Figure 3C,D) and genistein (Figure 3E,F), also indicating that daidzein and genistein’s enhancer effect on *Slc2a4* gene expression is mediated by ESR1, and not modulated by ESR2, at the concentration of 10 nM.

### 3.4. High Concentration of Daidzein and Genistein Reverses Their Enhancer Effect on the Slc2a4/GLUT4 Expression in Adipocytes

**Figure 4:**
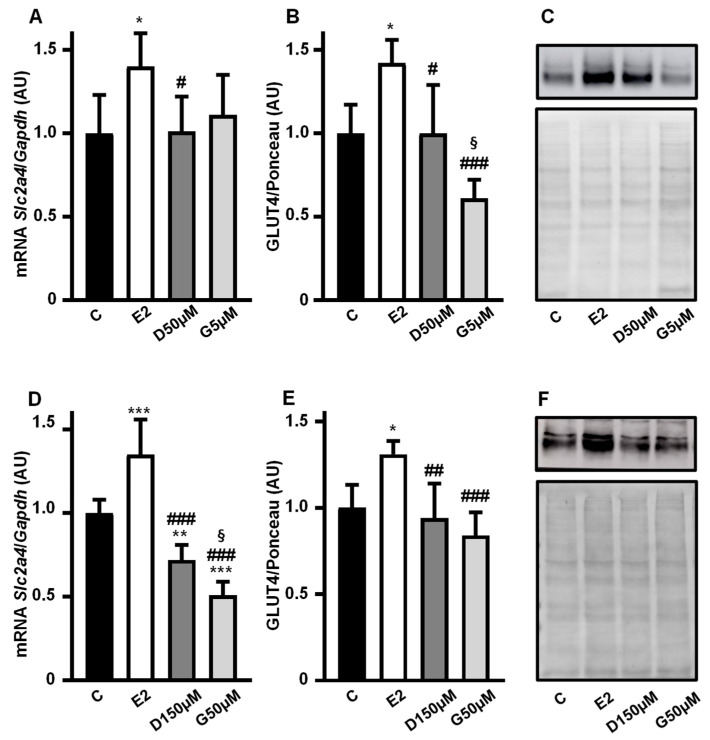
High concentrations of daidzein and genistein reverse the enhancer effect of phytoestrogen on the *Slc2a4*/GLUT4 expression in adipocytes. The *Slc2a4* mRNA (**A**,**D**) and GLUT4 protein (**B**,**C**,**E**,**F**) expressions were analyzed in differentiated 3T3-L1 adipocytes cultivated, for 24 h, with 10 nM 17β-estradiol, 50 µM and 150 µM daidzein and 5 µM and 50 µM genistein, as indicated in the graphics, and compared with a control treatment without hormone. The *Slc2a4* mRNA was analyzed by RT-qPCR, using the *Gapdh* mRNA as a loading control; the GLUT4 protein was quantified by Western blotting, using the Ponceau-stained membrane as a loading control. Representative blots of GLUT4 and respective Ponceau-stained membrane are shown in (**C**,**F**). Results were normalized considering the mean of C values as 1.0. Data are expressed as mean ± SD of 6 (panels (**A**,**B**,**D**)) or 5 (panel (**E**)) samples, from at least 3 different batches of experiments. The means were compared by one-way ANOVA, followed by Tukey’s multiple comparison test (* *p* < 0.05, ** *p* < 0.01, *** *p* < 0.001 vs. C; # *p* < 0.05, ## *p* < 0.01, ### *p* < 0.001 vs. E2; § *p* < 0.05 vs. D). C, control; E2, 17β-estradiol; D, daidzein; G, genistein; AU, arbitrary units; *Slc2a4*, solute carrier family 2 member 4; *Gapdh*, glyceraldehyde-3-phosphate dehydrogenase; GLUT4, solute carrier family 2, facilitated glucose transporter member 4.

*Slc2a4* mRNA (Figure 4A,D) and GLUT4 protein (Figure 4B,C,E,F) expressions were analyzed in 3T3-L1 adipocytes cultivated with 10 nM 17β-estradiol, 50 µM and 150 µM daidzein and 5 µM and 50 µM genistein for 24 h, and compared with a control treatment without hormone.

The enhancing effect on the *Slc2a4* mRNA expression disappeared at intermediary doses of 50 µM D and 5 µM G (Figure 4A), as did the increase in the GLUT4 protein (Figure 4B,C). When the maximal doses of 150 µM D and 50 µM G were evaluated, a significant decrease in *Slc2a4* mRNA (Figure 4D) was observed as compared to 10 nM E2 (*p* < 0.001) and even as compared to C (by 26%, *p* < 0.01 and by 44%, *p* < 0.001 for D and G, respectively). However, regarding the protein (Figure 4E,F), as compared to C, the reduction in response to D (by 7%) and G (by 16%) was not statistically significant.

Images of the original experiments from which the representative images of GLUT4 were selected, showing the entire Ponceau-stained membrane and the immunoblotted membrane, are presented as Supplementary Figure S2.

### 3.5. The Repressor Effect of High Concentrations of Daidzein and Genistein on the Slc2a4 Gene Expression in Adipocytes Seems to Involve ESR1 Inhibition and ESR2 Activation

**Figure 5:**
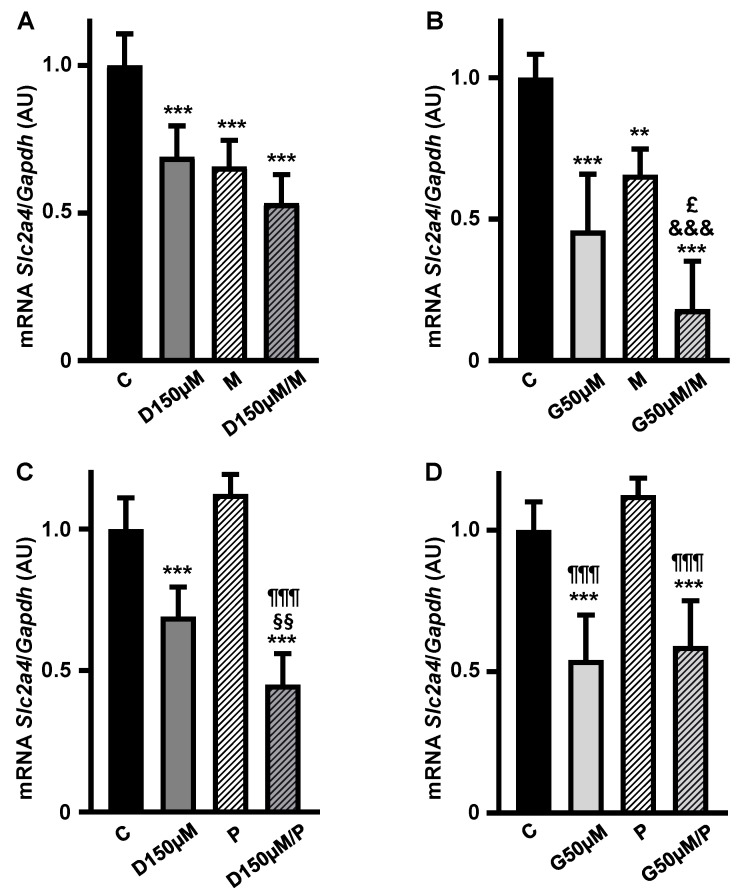
The repressor effect of high concentrations of daidzein and genistein on *Slc2a4* gene expression in adipocytes seems to involve ESR1 inhibition and ESR2 activation. The *Slc2a4* mRNA expression was analyzed in differentiated 3T3-L1 adipocytes cultivated, for 24 h, with 150 µM daidzein (**A**,**C**) and 50 µM genistein (**B**,**D**), combined or not with 1 µM ESR1 antagonist MPP (1,3-Bis(4-hydroxyphenyl)-4-methyl-5-[4-(piperidinylethoxy)phenol]-1H-pyrazol dihydrochloride) (**A**,**B**) or 1 µM ESR2 antagonist PHTPP (4-[2-Phenyl-5,7-bis(trifluoromethyl)pyrazolo[1,5-a]pyrimidin-3-yl]phenol) (**C**,**D**), and compared with a control treatment without hormone. The *Slc2a4* mRNA was analyzed by RT-qPCR, using the *Gapdh* mRNA as a loading control. Data are expressed as mean ± SD of 6 samples, from at least 3 different batches of experiments. The means were compared by one-way ANOVA, followed by Tukey’s multiple comparison test (** *p* < 0.01, *** *p* < 0.001 vs. C; §§ *p* < 0.01 vs. D; £ *p* < 0.05 vs. G; &&& *p* < 0.001 vs. M; ¶¶¶ *p* < 0.001 vs. P). C, control; D, daidzein; G, genistein; M, ESR1 antagonist MPP; P, ESR2 antagonist PHTPP; AU, arbitrary units; *Slc2a4*, solute carrier family 2 member 4; *Gapdh*, glyceraldehyde-3-phosphate dehydrogenase.

To investigate the participation of ESRs in the repressor effect of high doses of D and G, the addition of MPP (M) and PPTPP (P) antagonists to the maximal doses of 150 µM D (Figure 5A,B) and of 50 µM G (Figure 5C,D) was tested.

Inhibition of ESR1 in D- and G-treated cells (Figure 5A,B) promoted further reductions in the *Slc2a4* mRNA expression by 23% (*p* = 0.0225, Student *t* test) and 60% (*p* < 0.05, Student *t* test) as compared to D or G alone, respectively. These results suggest the inhibition of some residual activity of ESR1 and/or the activation of the ESR2 repressor effect.

On the other hand, addition of ESR2 antagonist (P) promoted a further reduction in the repressor effect of D alone (Figure 5C), whereas it did not alter the repressor effect of G; both results make it difficult to interpret the participation of ESR2 in the D and G repression of the *Slc2a4* gene expression.

## 4. Discussion

For many years it has been proposed that phytoestrogens have some beneficial effect on several diseases, including diabetes mellitus (DM) and obesity [24]. However, most of these studies do not explore the molecular mechanisms involved in the estrogenic and/or anti-estrogenic effects [24,25,26].

Although the participation of isoflavones in glycemic homeostasis control may involve several territories related to this regulation (mainly liver, skeletal muscle and adipose tissue), most investigations focus on adipose tissue, where phytoestrogens seem to play an additional role in adipogenesis, and, therefore, in obesity [26]; because of this, this study was performed in adipocytes. To the best of the authors’ knowledge, this is the first study investigating the effect of daidzein and genistein on the expression of the *Slc2a4* gene in differentiated 3T3L1 adipocytes, as well as the participation of estrogen receptors ESR1 and ESR2 in the effects observed. The results, in adipocytes, show that daidzein and genistein have a biphasic ESR1-mediated effect: while low concentrations increase the expression of *Slc2a4*/GLUT4, high concentrations decrease it, the former predisposing to an adipogenic effect, the latter to a diabetogenic condition.

E2 at a 10 nM concentration was chosen for comparison with daidzein (D) and genistein (G) because other studies from our group have been performed with this E2 concentration, reporting its positive effect on *Slc2a4*/GLUT4 expression and glucose uptake in adipocytes (for a review see [19]). Regarding concentrations of D and G, we started with the equimolar (10 nM) concentration, and thus higher concentrations were investigated, as will be discussed next. Furthermore, the GLUT4 protein was measured in a total cellular membrane protein fraction, obtained from fragments of plasma membrane and high- and low-density microsomes (the subcellular location of GLUT4), pelleted after differential centrifugation [23]. Because of this, proteins usually used as loading control for immunoblot, such as actin B, tubulin and GAPDH, are not expected to be present in stable quantities to be effectively used as a loading control. Thus, the optical density of the Ponceau-stained lane was used for normalizing the GLUT4 values, highlighting that the total amount of protein loaded in electrophoresis has long been considered the best internal control for Western blotting [27,28,29,30,31]. Furthermore, as original images of the representative GLUT4 blots showed in the figures, we are providing (in Supplementary Figures S1 and S2) the 40 to 70 KDa segment of the immunoblotted membrane, with a reminder that GLUT4 weighs 50–55 KDa.

At a low concentration (10 nM), D and G enhanced *Slc2a4*/GLUT4 expression, as did E2; however, this agonist effect was abolished in the presence of E2, thus suggesting that the interaction between D and G with E2 triggers an antagonist outcome, as already described for other effects [21,22]. In contrast, the GLUT4 expression in response to G or D plus E2 remained the same as when they were used in isolation, which may be due to the activation of some post-transcriptional regulations. Epigenetic mechanisms, such as some micro-RNAs and histone post-translational modifications, have already been described to modulate the *Slc2a4*/GLUT4 expression under diabetes-related conditions [32], explaining the dissociation between mRNA and protein regulations. In addition, variations in the *Slc2a4* mRNA poly(A) tail length, which modulates translation efficiency, had also been reported to occur in *Slc2a4* mRNA, also explaining discrepances between regulations of *Slc2a4* mRNA and GLUT4 protein [33]. Because the longer the poly(A) tail is, the more stable the mRNA is and the more efficient its translation is [33], here we could expect that the same amount of mRNA, but with an elongated poly(A) tail, would improve the mRNA translation, thus increasing the protein content. Finaly, we must consider that every reduction in protein expression depends on the half-life of the protein, which can reach up to 40 h for GLUT4 [34], with a reminder that we are analyzing 24 h regulations. The discrepancy between *Slc2a4* mRNA and GLUT4 protein variations observed is a limitation of this study.

Regarding the participation of ESRs in *Slc2a4* gene expression, MPP revealed that the positive enhancer effect of D and G, at 10 nM, is mediated by ESR1; in addition, the reduction in the *Slc2a4* mRNA expression observed in response to MPP alone indicated that ESR1 has positive constitutive transcriptional activity, as has already been described for other E2 target genes [9,10,11] and also for the *Slc2a4* gene [14,16]. On the other hand, the results with PHTPP indicated that ESR2 did not participate in the *Slc2a4* increase with neither E2 nor D and G (at 10 nM), but the increasing effect of PHTPP alone reveals that ESR2 displays a constitutive repressor effect on the *Slc2a4* gene, which was turned off in the presence of the antagonist, as already suggested [14].

Throughout the study, we investigated whether the enhancer effect of 10 nM D and 10 nM G would persist at increasing concentrations. For that, we drew on a study which evaluated the capacity of E2, D and G to induce the ESR1 or ESR2 binding and activation of a 70 bp ERE consensus sequence [20]. From the most to the least effective compound, the EC (50) for both ESR1 and ESR2 was described as E2 > G > D [19]. Since adipocytes preponderantly express ESR1 [12,13], our choices of D and G concentrations were based on their activity in response to ESR1, which was 10,000-fold and 500-fold lower for D and G, respectively, as compared to E2 [20]. Based on this, we should increase the concentrations of D and G to 100 µM and 5 µM, respectively, to equate their ESR1 activity to that of 10 nM E2.

Thus, doses of 50 µM D and 5 µM G were investigated, and, at these concentrations, the enhancer effect of 10 nM D and 10 nM G on the expression of *Slc2a4*/GLUT4 was completely abrogated. Therefore, the maximal concentrations of 150 µM D and 50 µM G were investigated, and these concentrations inhibited the *Slc2a4* mRNA expression by 3T3L1 adipocytes, indicating that isoflavones at these highest doses not only lose their capacity to activate ESR1, but also inhibit the constitutive positive effect on ESR1 (independent of ligand). In contrast, the magnitude of repression observed in the protein was smaller, probably involving some post-transcriptional regulation as discussed above, again with a reminder that reductions in the protein contents are always dependent on the half-life of the protein [32] and that this test was for gene expression and not for protein expression. D- and G-induced reduction in GLUT4 expression has already been suggested, also after 24 h of treatment with high doses of D and G; however, that study was performed in androgen-sensitive prostate cancer cells (LNCaP-R) and did not investigate *Slc2a4* gene expression [35].

The participation of ESR1 and ESR2 was also investigated in the 150 µM D- and 50 µM G-induced repression of *Slc2a4* gene expression. In the presence of D, MPP maintained the repressor effect of the isoflavone; however, in the presence of G, MPP promoted an additional stronger reduction in the *Slc2a4* mRNA expression, indicating that higher doses of isoflavones inhibit not only the ESR1-mediated enhancer effect on the *Slc2a4* gene, but also the constitutive enhancer effect on the gene; additionally, they could activate an ESR2-mediated repressor effect, as observed with 50 µM G. Regarding the ESR2 inhibitor PHTPP, alone, it showed a tendency towards an increase in the *Slc2a4* mRNA expression, which once more can be attributed to the inhibition of a small constitutive ESR2 repressor effect on the gene. However, when associated with 150 µM D or 50 µM G, instead of an expected possible increase in the *Slc2a4* mRNA, PHTPP induced an additional decrease in the gene expression, as observed with 150 µM D. These bizarre responses observed with PHTPP may be due to several problems: (1) the efficacy of the dose of PHTPP used; (2) the possibility that D and G, similarly to E2, trigger transactivation mechanisms of gene transcription which can have inhibitory or stimulatory activities [19]; and (3) variable indirect effects of the ESR ligands on monomeric forms of ESR1 and ESR2, which are present in the regulatory sequence of the *Slc2a4* gene, and are only partially characterized for E2 activity [19]. In sum, the possible participation of ESR2 in the repressor effect of high doses of D and G on *Slc2a4* gene expression is still unclear, deserving further additional investigations.

One important point of the present study is the dose-related biphasic effects of D and G observed, indicating a change from a pro-glucose consumption situation (at low dose) to a diabetogenic condition (at high dose), highlighting the relevance of invariably considering the D or G concentration before proposing some effect related to glycemic homeostasis. Importantly, some studies in this field have regularly used high doses of isoflavones; for instance, 25 µM of D or G was described to inhibit lipid accumulation in 3T3-L1 adipocytes [36], and G was reported to progressively inhibit some effects of insulin on rat adipocytes, by a post-receptor-mediated mechanism, achieving a complete blockade of insulin-induced glucose oxidation at 370 µM G [37]. On the other hand, in the pre-osteoblastic cell line KS483, G inhibited adipogenesis at low doses while stimulating adipogenesis at high doses [26], pointing out that KS483 is not an adipocyte. In addition, activation of proadipogenic mechanisms was recently reported in adipose tissue of genistein-supplemented mice [38].

Additional considerations are warranted based on the biological effects of isoflavone metabolites, especially those generated from isoflavone-rich foods [39]. Regarding D and G, all derivatives revealed ESR1-mediated biological activity like that of their precursors, with genistein and derivatives showing greater capacity to shift the ESR1 binding [40]. Thus, we can expect that the in vitro results of D and G described here may occur similarly in vivo, independently of their metabolization. Still, regarding the plasmatic kinetics of isoflavones obtained from soy-rich diets, other issues should be pointed out. In men, a single soy-rich meal increase isoflavone concentrations slowly, reaching maximum values of ~3 and ~4 µM, respectively, for D and G, between 7 and 8 h post meal [41]. In postmenopausal women, a traditional diet supplemented with soya flour provides plasma concentrations of 1.23 and 0.55 µM, respectively, for D and G [42]. Finally, in a review, a summary of genistein pharmacokinetics in response to oral intake in humans reported that the maximal plasma concentration is variable, according to the source and dose, several times achieving levels ≥5 µM [43]. These higher levels of isoflavones, regardless of their in vivo metabolites, may no longer promote beneficial effects regarding *Slc2a4*/GLUT4 expression.

Finally, considering the possible harmful effects of isoflavones described here, which may contribute to impairing metabolic homeostasis in vivo, some relevant systematic reviews and meta-analyses of isoflavone supplementation must be commented on. One study included 1687 women from 18 to 75 years of age (combining young adult women with postmenopausal women), supplemented with isolated or mixed isoflavones for 4 to 15 years; the study concluded that some isolated compounds could be beneficial to prevent DM, but not mixed isoflavones [44]. However, regarding DM prevention, several biases compromise the results of that study, such as variations in the age and estrogen blood levels of the participants, as well as in the length of the study. Another study in postmenopausal women supplemented with 40–150 mg/day of isoflavones for 1–12 months revealed that the consumption of isoflavones resulted in a significant reduction in triacylglycerol (TG) and a modest increase in high-density lipoprotein cholesterol (HDL) concentrations; however, analyses of subgroups revealed that isoflavones significantly decreased TG and increased HDL cholesterol only in participants under the age of 65; although both low (<80 mg/day) and high (>80 mg/day) doses of isoflavones have promoted TG-lowering effects, only the high dose increased HDL cholesterol [45]. This analysis points out that some effects of the in vivo supplementation with isoflavone also display a variable response, as described in the present study.

## 5. Conclusions

In sum, our study reveals that the isoflavones daidzein and genistein can have a dose-related biphasic effect on the expression of the Slc2a4 gene and GLUT4 protein in adipocytes, which must have important consequences regarding the capacity of glucose uptake by adipose tissue. Regarding this gene in adipocytes, this study shows that a low concentration of isoflavones favors a proadipogenic condition, while a high concentration favors a diabetogenic condition. Moreover, this study shows that the concentration of isoflavones must be always considered in investigations on their biological effects.

## Data Availability

The original contributions presented in this study are included in the article/Supplementary Materials. Further inquiries can be directed to the corresponding author.

## Supplementary Materials

The supporting information can be downloaded at https://www.mdpi.com/article/10.3390/cimb47100857/s1.

## Abbreviations

The following abbreviations are used in this manuscript:

ANOVA	one-way analysis of variance
AU	arbitrary units
D	daidzein
DM	diabetes mellitus
DMEM	Dulbecco’s modified eagle medium
DMSO	dimethylsulfoxide
FBS	fetal bovine serum
ECL	enhanced chemiluminescence
Erα	estrogen receptor alpha
Erβ	estrogen receptor beta
ERE	estrogen responsive element
ESR1	estrogen receptor 1
ESR2	estrogen receptor 2
E2	17β-estradiol
G	genistein
*Gapdh*	glyceraldehyde-3-phosphate dehydrogenase (mouse gene)
GLUT4	solute carrier family 2, facilitated glucose transporter member 4
HDL	high-density lipoprotein cholesterol
M	MPP (ESR1 antagonist)
MTT	tetrazolium salt 3-(4,5-dimethylthiazol-2-yl)-2,5-diphenyltetrazolium bromide
P	PHTPP (ESR2 antagonist)
RT-qPCR	reverse transcription and quantitative polymerase chain reaction
SD	standard deviation
*Slc2a4*	solute carrier family 2 member 4 (mouse gene)
TG	triacylglycerol

## References

[1] Rathery F., Rudolf M. (1928). Folliculine, insuline et diabète. Bull. Mem. Soc. Med. Hop. Paris.

[2] Sun L., Wang Y., Zhou T., Zhao X., Wang Y., Wang G., Gang X. (2019). Glucose metabolism in turner syndrome. Front. Endocrinol..

[3] Paschou S.A., Marina L.V., Spartalis E., Anagnostis P., Alexandrou A., Goulis D.G., Lambrinoudaki I. (2019). Therapeutic strategies for type 2 diabetes mellitus in women after menopause. Maturitas.

[4] Zeng X., Xie Y.J., Liu Y.T., Long S.L., Mo Z.C. (2020). Polycystic ovarian syndrome: Correlation between hyperandrogenism, insulin resistance and obesity. Clin. Chim. Acta.

[5] Cooke P.S., Nanjappa M.K., Ko C., Prin G.S., Hess R.A. (2017). Estrogens in male physiology. Physiol. Rev..

[6] Faustini-Fustini M., Rochira V., Carani C. (1999). Oestrogen deficiency in men: Where are we today?. Eur. J. Endocrinol..

[7] Belgorosky A., Guercio G., Pepe C., Saraco N., Rivarola M.A. (2009). Genetic and clinical spectrum of aromatase deficiency in infancy, childhood and adolescence. Horm. Res..

[8] Elbers J.M.H., Giltay E.J., Teerlink T., Scheffer P.G., Asscheman H., Seidell J.C., Gooren L.J.G. (2003). Effects of sex steroids on components of the insulin resistance syndrome in transsexual subjects. Clin. Endocrinol..

[9] Heldring N., Pike A., Andersson S., Matthews J., Cheng G., Hartman J., Tujague M., Ström A., Treuter E., Warner M. (2007). Estrogen receptors: How do they signal and what are their targets. Physiol. Rev..

[10] Nilsson S., Mäkelä S., Treuter E., Tujague M., Thomsen J., Andersson G., Enmark E., Pettersson K., Warner M., Gustafsson J.Å. (2001). Mechanisms of Estrogen Action. Physiol. Rev..

[11] Safe S., Kim K. (2008). Non-classical genomic estrogen receptor (ER)/specificity protein and ER/activating protein-1 signaling pathways. J. Mol. Endocrinol..

[12] Barros R.P.A., Machado U.F., Warner M., Gustafsson J.A. (2006). Muscle GLUT4 regulation by estrogen receptors ERbeta and ERalpha. Proc. Natl. Acad. Sci. USA.

[13] Barros R.P.A., Gustafsson J.A. (2011). Estrogen receptors and the metabolic network. Cell Metab..

[14] Campello R.S., Alves-Wagner A.B., Lucas T.F., Mori R.C., Furuya D.T., Porto C.S., Machado U.F. (2012). Estrogen receptor 1 agonist PPT stimulates *Slc2a4* gene expression and improves insulin-induced glucose uptake in adipocytes. Curr. Top. Med. Chem..

[15] Klip A., McGraw T.E., James D.E. (2019). Thirty sweet years of GLUT4. J. Biol. Chem..

[16] Campello R.S., Fátima L.A., Barreto-Andrade J.N., Lucas T.F., Mori R.C., Porto C.S., Machado U.F. (2017). Estradiol-induced regulation of GLUT4 in 3T3-L1 cells: Involvement of ESR1 and AKT activation. J. Mol. Endocrinol..

[17] Gupta C., Prakash D., Gupta S. (2016). Phytoestrogens as pharma foods. Adv. Food Technol. Nutr. Sci. Open J..

[18] Pilšáková L., Riečanský I., Jagla F. (2010). The physiological actions of isoflavone phytoestrogens. Physiol. Res..

[19] Gregorio K.C.R., Laurindo C.P., Machado U.F. (2021). Estrogen and Glycemic Homeostasis: The Fundamental Role of Nuclear Estrogen Receptors ESR1/ESR2 in Glucose Transporter GLUT4 Regulation. Cells.

[20] Kostelac D., Rechkemmer G., Briviba K. (2003). Phytoestrogens Modulate Binding Response of Estrogen Receptors α and β to the Estrogen Response Element. J. Agric. Food Chem..

[21] Turner J.V., Glass B.D., Agatonovic-Kustrin S. (2007). Molecular aspects of phytoestrogen selective binding at estrogen receptors. J. Pharm. Sci..

[22] Kuiper G.G., Lemmen J.G., Carlsson B., Corton J.C., Safe S.H., van der Saag P.T., van der Burg B., Gustafsson J.A. (1998). Interaction of estrogenic chemicals and phytoestrogens with estrogen receptor beta. Endocrinology.

[23] Pinto-Junior D.C., Silva K.S., Michalani M.L., Yonamine C.Y., Esteves J.V., Fabre N.T., Thieme K., Catanozi S., Okamoto M.M., Seraphim P.M. (2018). Advanced glycation end products-induced insulin resistance involves repression of skeletal muscle GLUT4 expression. Sci. Rep..

[24] Bhathena S.J., Velasquez M.T. (2002). Beneficial role of dietary phytoestrogens in obesity and diabetes. Am. J. Clin. Nutr..

[25] Pandozzi C., Giannetta E., Tarsitano M.G. (2022). Phytotherapic approach in menopause: Light and darkness. Minerva Endocrinol..

[26] Dang Z.C. (2009). Dose-dependent effects of soy phyto-oestrogen genistein on adipocytes: Mechanisms of action. Obes. Rev..

[27] Klein D., Kern R.M., Sokol R.Z. (1995). A method for quantification and correction of proteins after transfer to immobilization membranes. Biochem. Mol. Biol. Int..

[28] Ferguson R.E., Carroll H.P., Harris A., Maher E.R., Selby P.J., Banks R.E. (2005). Housekeeping proteins: A preliminary study illustrating some limitations as useful references in protein expression studies. Proteomics.

[29] Romero-Calvo I., Ocón B., Martínez-Moya P., Suárez M.D., Zarzuelo A., Martínez-Augustin O., de Medina F.S. (2010). Reversible Ponceau staining as a loading control alternative to actin in Western blots. Anal. Biochem..

[30] Welinder C., Ekblad L. (2011). Coomassie Staining as Loading Control in Western Blot. J. Proteome Res..

[31] Moritz C.P. (2017). Tubulin or not tubulin: Heading toward total protein staining as loading control in Westernblots. Proteomics.

[32] Esteves J.V., Yonamine C.Y., Machado U.F. (2020). *SLC2A4* expression and its epigenetic regulation as biomarkers for insulin resistance treatment in diabetes mellitus. Biomark. Med..

[33] Seraphim P.M., Nunes M.T., Giannocco G., Machado U.F. (2007). Age related obesity-induced shortening of GLUT4 mRNA poly(A) tail length in rat gastrocnemius skeletal muscle. Mol. Cell. Endocrinol..

[34] Shi J., Kandror K.V. (2005). Sortilin is essential and sufficient for the formation of Glut4 storage vesicles in 3T3-L1 adipocytes. Dev. Cell.

[35] Gonzalez-Menendez P., Hevia D., Rodriguez-Garcia A., Mayo J.C., Sainz R.M. (2014). Regulation of GLUT transporters by flavonoids in androgen-sensitive and -insensitive prostate cancer cells. Endocrinology.

[36] Tan J., Huang C., Luo Q., Liu W., Cheng D., Li Y., Xia Y., Li C., Tang L., Fang J. (2019). Soy Isoflavones Ameliorate Fatty Acid Metabolism of Visceral Adipose Tissue by Increasing the AMPK Activity in Male Rats with Diet-Induced Obesity (DIO). Molecules.

[37] Abler A., Smith J.A., Randazzo P.A., Rothenberg P.L., Jarett L. (1992). Genistein differentially inhibits postreceptor effects of insulin in rat adipocytes without inhibiting the insulin receptor kinase. J. Biol. Chem..

[38] Kwon H., Han H., Oh Y., Kim Y., Kim J.H. (2025). Anti-cancer effects of genistein supplementation and moderate-intensity exercise in high-fat diet-induced breast cancer via regulation of inflammation and adipose tissue metabolism in vivo and in vitro. BMC Complement. Med. Ther..

[39] Adlercreutz H., Fotsis T., Lampe J., Wähälä K., Mäkelä T., Brunow G., Hase T. (1993). Quantitative Determination of Lignans and Isoflavonoids in Plasma of Omnivorous and Vegetarian Women by Isotope Dilution Gas Chromatographγ-Mass Spectrometry. Scand. J. Clin. Lab. Investig..

[40] Kinjo J., Tsuchihashi R., Morito K., Hirose T., Aomori T., Nagao T., Okabe H., Nohara T., Masamune Y. (2004). Interactions of phytoestrogens with estrogen receptors alpha and beta (III). Estrogenic activities of soy isoflavone aglycones and their metabolites isolated from human urine. Biol. Pharm. Bull..

[41] King R., Bursill D. (1998). Plasma and urinary kinetics of the isoflavones daidzein and genistein after a single soy meal in humans. Am. J. Clin. Nutr..

[42] Morton M.S., Wilcox G., Wahlqvist M.L., Griffiths K. (1998). Determination of lignans and isoflavonoids in human female plasma following dietary supplementation. J. Endocrinol..

[43] Yang Z., Kulkarni K., Zhu W., Hu M. (2012). Bioavailability and pharmacokinetics of genistein: Mechanistic studies on its ADME. Anti-Cancer Agents Med. Chem..

[44] Glisic M., Kastrati N., Musa J., Milic J., Asllanaj E., Fernandez E.P., Nano J., Rosales C.O., Amiri M., Kraja B. (2018). Phytoestrogen supplementation and body composition in postmenopausal women: A systematic review and meta-analysis of randomized controlled trials. Maturitas.

[45] Yang S., Zeng Q., Huang X., Liang Z., Hu H. (2023). Effect of Isoflavones on Blood Lipid Alterations in Postmenopausal Females: A Systematic Review and Meta-Analysis of Randomized Trials. Adv. Nutr..

