# Supplementary material for: Biphasic Slc2a4 Gene Expression in 3T3-L1 Adipocytes in Response to Treatment with Low and High Concentrations of Daidzein and Genistein
Source: Curr Issues Mol Biol. 2025 Oct 17;47(10):857. doi: 10.3390/cimb47100857 (PMC12564231; doi:10.3390/cimb47100857)

Supplementary Figure S1

Figure 2 C

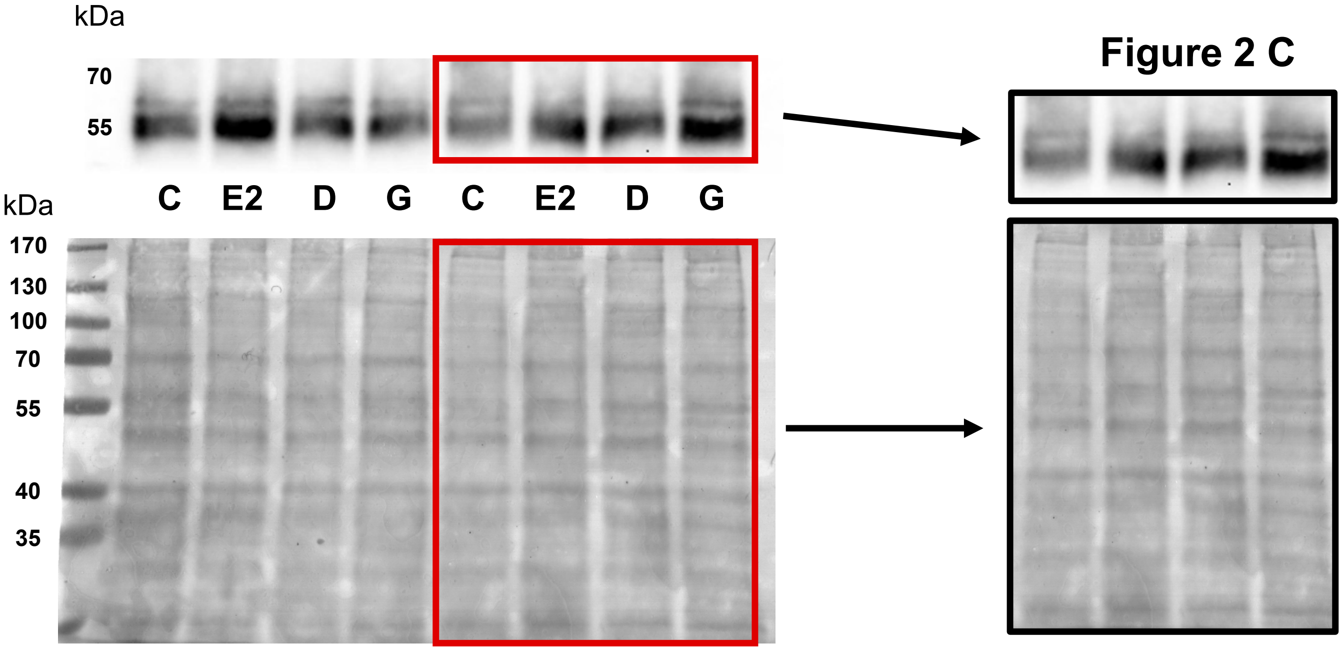

Figure 2 F

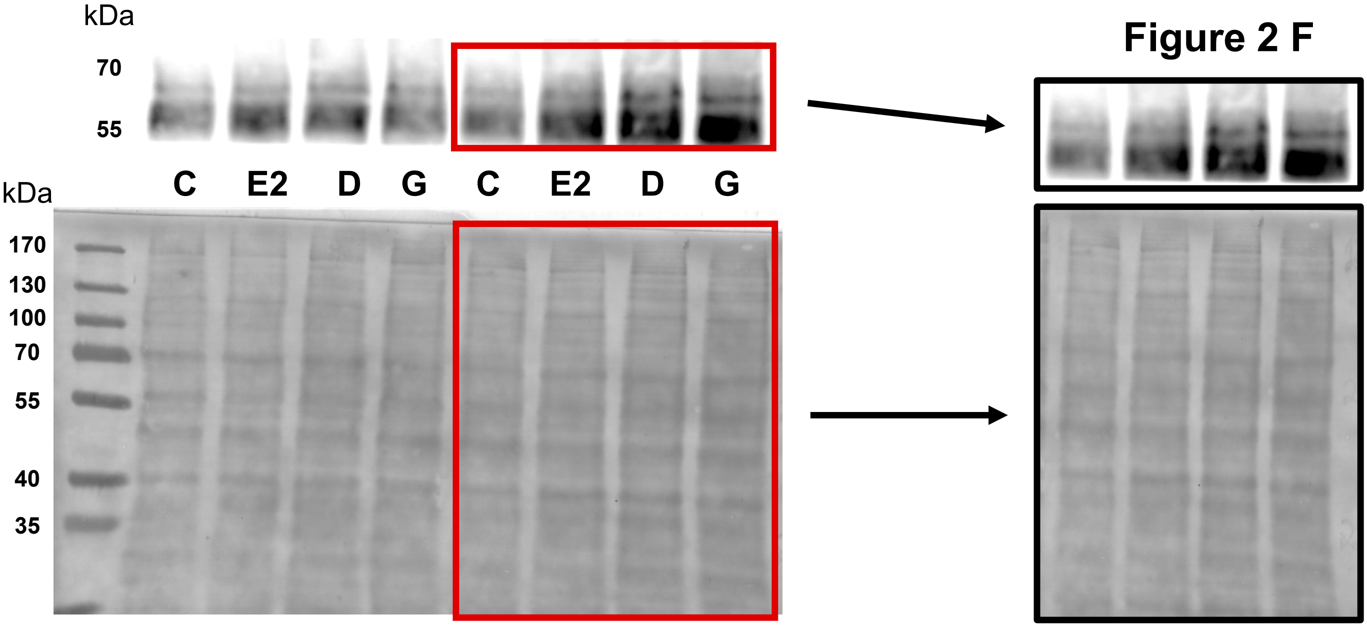

Supplementary Figure S2

Figure 4 C

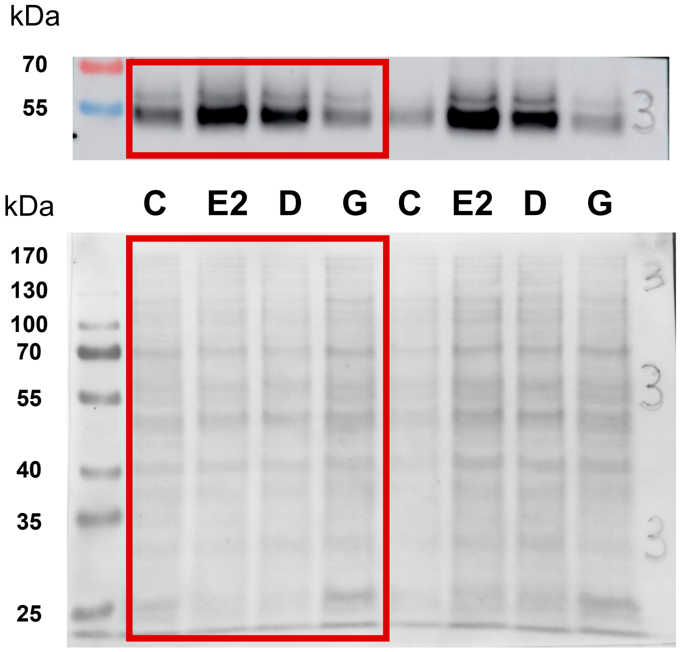

Figure 4 C

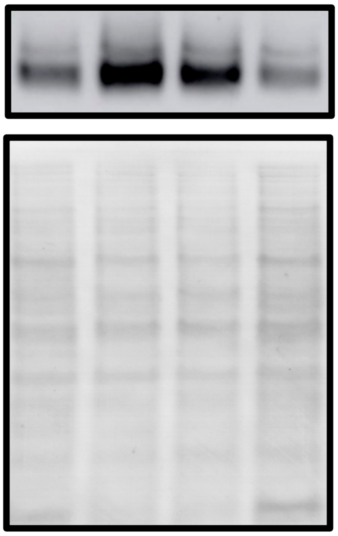

Figure 4 F

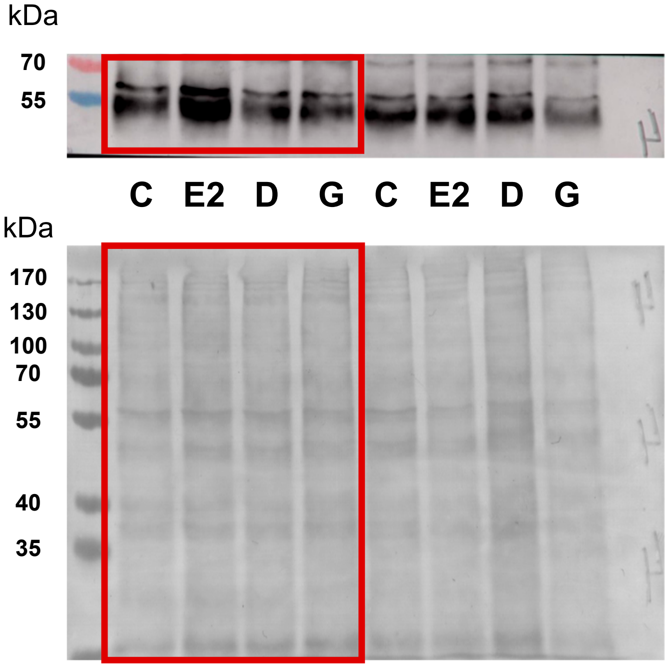

Figure 4 F

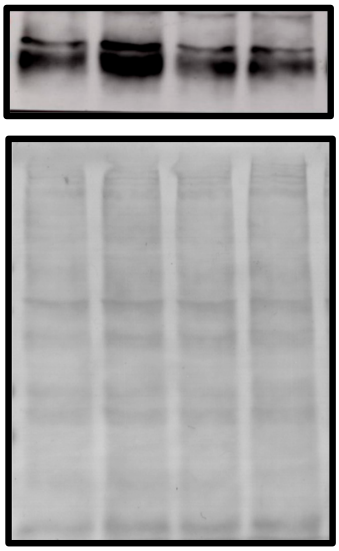

Supplement: Supplementary file 1 [file cimb-47-00857-s001.zip › cimb-3908672-supplementary.pdf]
